# Pseudo-scalar Higgs boson production at N$^3$LO$_{\text {A}}$+N$^3$LL$'$

**DOI:** 10.1140/epjc/s10052-016-4510-1

**Published:** 2016-12-01

**Authors:** Taushif Ahmed, Marco Bonvini, M. C. Kumar, Prakash Mathews, Narayan Rana, V. Ravindran, Luca Rottoli

**Affiliations:** 1 The Institute of Mathematical Sciences, IV Cross Road CIT Campus, Taramani, Chennai, 600113 India; 2 Rudolf Peierls Centre for Theoretical Physics, University of Oxford, 1 Keble Road, Oxford, OX1 3NP UK; 3 Department of Physics, Indian Institute of Technology Guwahati, Guwahati, 781039 India; 4 Saha Institute of Nuclear Physics, 1/AF Bidhan Nagar, Kolkata, 700064 India

## Abstract

We consider the production of a pseudo-scalar particle *A* at the LHC, and present accurate theoretical predictions for its inclusive cross section in gluon fusion. The prediction is based on combining fixed-order perturbation theory and all-order threshold resummation. At fixed order we include the exact next-to-next-to-leading order (NNLO) plus an approximate next-to-next-to-next-to-leading order (N$^3$LO$_\mathrm{A}$) which is based on the recent computation at this order for the scalar case. We then add threshold resummation at next-to-next-to-next-to leading logarithmic accuracy (N$^3$LL$^\prime$). Various forms of threshold resummation are considered, differing by the treatment of subleading terms, allowing a robust estimate of the theoretical uncertainties due to missing higher orders. With particular attention to pseudo-scalar masses of 200 and 750 GeV, we also observe that perturbative convergence is much improved when resummation is included. Additionally, results obtained with threshold resummation in direct QCD are compared with analogous results as computed in soft-collinear effective theory, which turn out to be in good agreement. We provide precise predictions for pseudo-scalar inclusive cross section at 13 TeV LHC for a wide range of masses. The results are available through updated versions of the public codes ggHiggs and TROLL.

## Introduction

The discovery of the Higgs boson by ATLAS [1] and CMS [2] collaborations of the Large Hadron Collider (LHC) at CERN has put the Standard Model (SM) of particle physics on a firmer ground. This has led to a better understanding of the dynamics behind the electroweak symmetry breaking [3–7]. In addition, the measured Higgs decay rates [8, 9] to $W^+ W^-$, *ZZ* and heavy fermion pairs are in good agreement with the predictions of the SM. Moreover, there are continuous efforts in the ongoing 13 TeV run at the LHC to establish Higgs quantum numbers such as spin and parity, even though there are already indications that it is a scalar with even parity [9, 10].

However, in spite of its spectacular success, it is well known that the SM fails to explain certain phenomena of the nature such as baryon asymmetry in the universe, the existence of the dark matter, the tiny non-zero mass of the neutrinos, etc. Explaining these phenomena requires one to go beyond the wall of the SM. Among the several existing models, supersymmetric theories provide an elegant solution to the aforementioned problems. In one of its simplest realisations, the minimal supersymmetric extension of the SM (MSSM), the Higgs sector contains two CP-even (scalar), one CP-odd (pseudo-scalar) and two charged Higgs bosons [11–18]. More generally, the existence of additional scalar and pseudo-scalar bosons which couple to fermions is a prediction of many models which include two Higgs doublets.

If we were to identify the lighter CP-even Higgs boson of these models with the observed scalar at the LHC [19–21], searches of other Higgs bosons become inevitable. In particular, for small and moderate $\tan \beta$ (the ratio of vacuum expectation values $v_1$ and $v_2$ of the two doublets), the large gluon flux at the LHC can provide an opportunity to search for other Higgs bosons. There are already efforts along this direction by the LHC collaborations. However, the experimental searches crucially depend on precise theoretical predictions. The goal of this work is to provide precise and accurate theoretical predictions for pseudo-scalar Higgs boson production.

The theoretical predictions for the production of a pseudo-scalar particle at the LHC have been already available up to NNLO level [22–24] in perturbative QCD in the heavy top-quark limit. These corrections are large, of the order of 67% at NLO and an additional 15% at NNLO at the central renormalisation and factorisation scale $\mu _\mathrm{R}=\mu _\mathrm{F}=m_A/2$, $m_A=200$ GeV. To achieve sufficient precision, the inclusion of higher orders is therefore necessary. This situation is very similar to that of scalar Higgs boson production, for which the N$^3$LO contribution is now known [25, 26]. This is further improved by the resummation of threshold logarithms, arising from soft-gluon emissions, to N$^3$LL$^\prime$ accuracy [27, 28],^1^ leading to a precise determination of the SM Higgs cross section at LHC with small and reliable uncertainty.

The computation of the N$^3$LO contribution to pseudo-scalar boson production in the threshold limit has been recently presented in Ref. [30]. In this article, we propose a new determination of the pseudo-scalar boson N$^3$LO cross section based on the recent result at this order for scalar production [26]. Then we study the inclusion of threshold resummation effects to pseudo-scalar production. We do this both in the standard formalism of direct QCD, as well as in the soft-collinear effective theory (SCET) approach. We find that the inclusion of threshold resummation together with the approximate N$^3$LO provides a significant increase of the precision for pseudo-scalar production and a marked reduction of the theoretical uncertainties. Our work extends previous results [31, 32] to the next fixed and logarithmic order in QCD.

The structure of this paper is the following. In Sect. 2 we introduce the notations and discuss fixed-order results for pseudo-scalar Higgs production. In Sect. 3 we give an overview of threshold resummation both in direct QCD and SCET, and present our strategy for the computation of the theoretical uncertainties. We describe how to construct a precise approximation of the pseudo-scalar Higgs cross section at N$^3$LO in Sect. 4. The numerical impact for pseudo-scalar Higgs production at LHC is presented in Sect. 5. We conclude in Sect. 6.

## Pseudo-scalar production

The inclusive cross section at hadron colliders with centre of mass energy $\sqrt{s}$ for the production of a colourless pseudo-scalar particle *A* of mass $m_A$ can be written as a convolution

1 $$\begin{aligned} \sigma (\tau , m_A^2) = \tau \sigma _0\sum _{i,j}\int _{\tau }^1\frac{\mathrm{d}z}{z} \mathscr {L}_{ij}\left( \frac{\tau }{z},\mu _{\scriptscriptstyle \mathrm F}^2\right) C_{ij}(z,\alpha _s,\mu _{\scriptscriptstyle \mathrm F}^2)\nonumber \\ \end{aligned}$$

of a perturbative partonic coefficient function $C_{ij}(z,\alpha _s,\mu _{\scriptscriptstyle \mathrm F}^2)$ and a parton luminosity

2 $$\begin{aligned} \mathscr {L}_{ij}\left( x,\mu _{\scriptscriptstyle \mathrm F}^2\right) = \int _{x}^1\frac{\mathrm{d}x'}{x'} f_i\left( \frac{x}{x'},\mu _{\scriptscriptstyle \mathrm F}^2\right) f_j\left( x',\mu _{\scriptscriptstyle \mathrm F}^2\right) , \end{aligned}$$

which is a convolution of parton distribution functions (PDFs) $f_i$ and $f_j$ of the initial state partons *i* and *j*, and $\tau =m_A^2/s$. For simplicity, we assume that $\alpha _s=\alpha _s(\mu _{\scriptscriptstyle \mathrm F}^2)$; computing $\alpha _s$ at a different renormalisation scale $\mu _{\scriptscriptstyle \mathrm R}$ and supplying the coefficients with the corresponding logarithms of the scale is a straightforward task. The prefactor $\sigma _0$, in the case the production is driven by just a top-quark loop with mass $m_t$, reads

3 $$\begin{aligned} \sigma _0&= \frac{\alpha _s^2 G_F}{32 \sqrt{2}\pi } \cot ^2\beta \,\big |x_t f(x_t)\big |^2, \qquad x_t = \frac{4m_t^2}{m_A^2},\end{aligned}$$

4 $$\begin{aligned} f(x_t)&= {\left\{ \begin{array}{ll} \mathrm{arcsin}^{2}\frac{1}{\sqrt{x_t}} &{}\quad x_t \ge 1\,, \\ -\frac{1}{4} \left( \ln \frac{1-\sqrt{1-x_t}}{1+\sqrt{1-x_t}} +i \pi \right) ^{2} &{}\quad x_t < 1, \end{array}\right. } \end{aligned}$$

and it is such that $C_{gg}$ is normalised to $\delta (1-z)$ at LO. In this equation, we assumed a Two Higgs Doublet Model with mixing angle $\beta$. In the following, we shall not make any assumption on $\beta$, and present results ignoring the $\cot ^2\beta$ term: the resulting cross sections can then be rescaled multiplying by $\cot ^2\beta$ to obtain a prediction for any desired value of $\beta$.

The coefficient functions $C_{ij}$ can be computed in perturbation theory. The NLO [33–36] and NNLO [22–24] QCD corrections to the coefficient functions are known in the large-$m_t$ effective theory, and the NLO also in the exact theory [36, 37]. Finite $1/m_t$ corrections at NNLO have been computed in Ref. [38]. Threshold contributions at N$^3$LO in the large-$m_t$ limit have been computed in Ref. [30], allowing for the computation of an approximate N$^3$LO prediction based on soft-virtual terms.

In this work, we propose a new way of approximating the N$^3$LO contribution, based on the recent result for scalar Higgs production in the large-$m_t$ effective theory, Ref. [26]. This approximation turns out to be much more precise than any soft-virtual approximation, and allows us to predict the N$^3$LO cross section for pseudo-scalar Higgs production up to corrections which we expect to be small. We describe our approximation in Sect. 4, after introducing the necessary ingredients in the next section.

## Threshold resummation

We now turn to briefly discussing threshold resummation. In this work we consider both the standard direct QCD (dQCD) approach  [39–43] and the soft-collinear effective theory (SCET) approach [44–47]. We refer the reader to [48–51] for a more detailed discussion of the comparison between the two frameworks.

Since threshold logarithmic enhancement affects only the gluon–gluon channels, from now on we will focus on the gluon fusion subprocess, and we will thus drop the parton indices *i*, *j* assuming they are both equal to *g*. Resummation (in dQCD) is usually performed in Mellin space, since the soft-gluon emission phase space factorises under Mellin transformation. The Mellin transformed cross section, Eq. (1), is given by

5 $$\begin{aligned} \sigma (N,m_A^2) \equiv \int _0^1 \mathrm{d}\tau \ \tau ^{N-2} \sigma (\tau ,m_A^2) = \sigma _0 \mathscr {L}(N) C(N,\alpha _s),\nonumber \\ \end{aligned}$$

where we have defined

6 $$\begin{aligned} \mathscr {L}(N)&\equiv \int _0^1 \mathrm{d}z\ z^{N-1} \mathscr {L}(z),\end{aligned}$$

7 $$\begin{aligned} C(N,\alpha _s)&\equiv \int _0^1 \mathrm{d}z \ z^{N-1} C(z, \alpha _s) \end{aligned}$$

and for simplicity we have suppressed the dependence on the factorisation scale $\mu _{\scriptscriptstyle \mathrm F}$.

In *N* space the threshold limit $z\rightarrow 1$ corresponds to the limit $N\rightarrow \infty$. All the non-vanishing contributions to the coefficient function $C(N,\alpha _s)$ can be computed using standard techniques developed long ago [39–43], and one can obtain the all-order resummed coefficient function

8 $$\begin{aligned} C_{N\text {-soft}} (N, \alpha _s) = g_0 (\alpha _s) \exp \mathcal S (\alpha _s,\ln N), \end{aligned}$$

where $g_0 (\alpha _s)$ is a power series in $\alpha _s$ and $\mathcal S (\alpha _s,\ln N)$ contains purely logarithmically enhanced terms. This result, which is the standard form of threshold resummation in dQCD, has been called *N*-soft in Ref. [27]. While the function $\mathcal {S}$ needed for N$^3$LL$^\prime$ accuracy has been known for a while [52], as it is identical for pseudo-scalar and scalar Higgs production, the constant function $g_0$ for pseudo-scalar production was known to second order [31, 32] and it has been computed to third order only recently [30].

Besides *N*-soft, there exist several prescriptions, formally equivalent in the large-*N* limit, which differ by either power suppressed 1 / *N* (subdominant) contributions or subleading logarithmic terms. We refer the reader to Ref. [27] for a more detailed discussion. In this work, we will use the approach of Ref. [28], where it is suggested to vary both subleading and subdominant contributions to estimate the impact of unknown higher-order terms. Specifically, following Ref. [28], we consider the so-called $\psi$-soft prescription, which essentially amounts to replacing $\ln N\rightarrow \psi _0(N)$ in the Sudakov exponent and performing a collinear improvement. The resulting default prescription, $\psi$-soft$_2$ (or $\psi$-soft AP2) [27, 28], is given by

9 $$\begin{aligned} C_{\psi \text {-soft}_2} (N, \alpha _s) = g_0 (\alpha _s) \exp&\Big [ 2\mathcal S (\alpha _s,\psi _0(N)) \nonumber \\&- 3 \mathcal S (\alpha _s,\psi _0(N+1)) \nonumber \\&+ 2 \mathcal S (\alpha _s,\psi _0(N+2))\Big ]. \end{aligned}$$

The linear combination of shifted exponents implements the collinear improvement AP2, obtained by retaining the LO splitting function $P_{gg}$ to second order in an expansion in $1-z$. Alternatively, one can keep only the first order (AP1), leading to

10 $$\begin{aligned} C_{\psi \text {-soft}_1} (N, \alpha _s) = g_0 (\alpha _s) \exp \mathcal S (\alpha _s,\psi _0(N+1)), \end{aligned}$$

which differs from $\psi$-soft$_2$ by subdominant 1 / *N* contributions. Subleading contributions are probed by moving some or all constant terms from $g_0$ to the exponent. This does not spoil the logarithmic accuracy of the result, but different subleading logarithmic contributions are generated by interference with the constant terms. The default position of the constant is determined by retaining in the exponent those constant terms that naturally arise there from Mellin transformation of threshold logarithms (see Ref. [28] for further details). The two variations correspond to either having all constants in the exponent, or no constants in the exponent; in the latter option all constants are in $g_0$, as in Eqs. (9), (10).

The approach of Ref. [28] consists then in computing the central value of the resummation according to $\psi$-soft$_2$ with the default option for the constants, and the uncertainty on this result from an envelope of scale variation, variation of 1 / *N* terms (AP1 vs. AP2) and variation of subleading terms (position of the constants). This rather conservative procedure for estimating the uncertainty has proved very powerful in the case of SM Higgs production, where higher-order corrections are large and fixed-order scale uncertainty is an unsatisfactory estimator of missing higher orders [28], at least for the first orders. As we shall see in the next Section, very similar results are found for pseudo-scalar production, which also suffers from large perturbative corrections.

Alternatively, soft-gluon resummation can be performed in the SCET framework [44–47]. In this formalism, the partonic coefficient function $C(z,\alpha _s,\mu _{\scriptscriptstyle \mathrm F}^2)$ is written in a factorised form as a result of a sequence of matching steps in which hard and soft modes are subsequently integrated out

11 $$\begin{aligned} C(z,\alpha _s,\mu _{\scriptscriptstyle \mathrm F}^2) = H(\mu _{\scriptscriptstyle \mathrm F}^2)\, S(z,\mu _{\scriptscriptstyle \mathrm F}^2), \end{aligned}$$

where $H(\mu _{\scriptscriptstyle \mathrm F}^2)$ and $S(z,\mu _{\scriptscriptstyle \mathrm F}^2)$ are known as hard function and soft function, respectively, and are given as power expansions in $\alpha _s$ computed at their last argument. While the soft function at N$^3$LO is the same for pseudo-scalar and scalar Higgs production and it has been known for a while [29, 53], the N$^3$LO hard function for pseudo-scalar production has been recently computed in Ref. [54].

The hard and soft functions satisfy renormalisation group equations in $\mu _{\scriptscriptstyle \mathrm F}$ that can be solved exactly. We can thus write the hard and soft functions in terms of a hard scale $\mu _{\scriptscriptstyle \mathrm H}$ and a soft scale $\mu _{\scriptscriptstyle \mathrm S}$, respectively, by introducing evolution factors which evolve them to the common scale $\mu _{\scriptscriptstyle \mathrm F}$:

12 $$\begin{aligned} C(z,\alpha _s,\mu _{\scriptscriptstyle \mathrm F}^2) = H(\mu _{\scriptscriptstyle \mathrm H}^2)\, S(z,\mu _{\scriptscriptstyle \mathrm S}^2)\, U(\mu _{\scriptscriptstyle \mathrm H}^2,\mu _{\scriptscriptstyle \mathrm S}^2,\mu _{\scriptscriptstyle \mathrm F}^2). \end{aligned}$$

The hard and soft scale should be chosen such that the perturbative expansions of *H* and *S* are well behaved. While for *H* $\mu _{\scriptscriptstyle \mathrm H}\sim m_A$, for the soft function a typically smaller scale, related to the scale of soft-gluon emission, is more appropriate. Therefore, the evolution *U* from $\mu _{\scriptscriptstyle \mathrm S}$ to $\mu _{\scriptscriptstyle \mathrm F}$ performs the resummation of the potentially large logarithms due to soft radiation. For the precise choice of scales, we follow the prescription of the original work [44].

In this work, we follow Ref. [29] and consider two independent variations of the SCET resummation: the variation of subleading 1 / *N* terms (corresponding in *z* space to $(1-z)^0$ terms), and the inclusion of the so-called $\pi ^2$-resummation [55–60]. As for dQCD, resumming $\pi ^2$ constant terms effectively changes subleading terms in the resummation. On the other hand, the variation of 1 / *N* terms is obtained through the inclusion of a collinear improvement, which effectively amounts to multiplying the soft function by an overall factor *z* [29]. This collinear improvement corresponds to the AP1 version of $\psi$-soft.

## Approximate N$^3$LO cross section

The recently computed SCET hard function *H* [54], together with the known soft function [29, 53], allowed the computation of all soft-virtual terms of N$^3$LO pseudo-scalar Higgs production [30], i.e. the plus distributions terms and the $\delta (1-z)$ term of the coefficient $C_{gg}$. The quality of such a soft-virtual approximation can be rather good as well as very poor. The reason is that the soft-virtual terms alone are defined only up to next-to-soft contributions, i.e. terms suppressed by at least one power of $(1-z)$ with respect to the soft ones, and these next-to-soft terms are usually quite significant [61–63]. Therefore, the quality of any soft-virtual approximation strongly depends on the control one has on the next-to-soft contributions. Moreover, the soft-virtual approximation only predicts the *gg* channel, while other partonic channels, which do not present logarithmic enhancement at threshold, cannot be predicted. However, other partonic channels, most importantly the *qg* channel, give a contribution which is non-negligible. Additionally, including all channels stabilises the factorisation scale dependence, which is instead unbalanced when only the *gg* channel is included.

In this work we exploit the similarity of pseudo-scalar Higgs production to scalar Higgs production to provide an approximation which overcomes all the limitations of a soft-virtual approximation. Calling $C^{H}_{ij}$ the coefficient functions for scalar Higgs production, we can write the coefficient functions for pseudo-scalar Higgs production as

13 $$\begin{aligned} C_{ij}(z,\alpha _s) = \frac{g_0(\alpha _s)}{g_0^H(\alpha _s)}\Big [C_{ij}^H(z,\alpha _s) + \delta C_{ij}(z,\alpha _s)\Big ], \end{aligned}$$

where $g_0(\alpha _s)$ is the constant function of dQCD resummation for pseudo-scalar Higgs, Eq. (8), and $g_0^H(\alpha _s)$ is the analogous function for scalar Higgs. Eq. (13) effectively defines $\delta C_{ij}(z,\alpha _s)$ as the correction to the scalar Higgs coefficient functions such that the rescaling $g_0/g_0^H$ converts them to the pseudo-scalar coefficients. Expanding order by order in $\alpha _s$ both sides of Eq. (13), the coefficients $\delta C_{ij}$ at $\mathcal {O}(\alpha _s^k)$ can be constructed from the knowledge of the scalar and pseudo-scalar coefficients $C_{ij}$ and $C^H_{ij}$ and of the constant functions $g_0$ and $g^H_0$ up to the same order. All ingredients are known up to NNLO, allowing the computation of $\delta C_{ij}$ at this order. At N$^3$LO, $g_0$ and $g^H_0$ are known from resummation [27, 30, 64, 65] and $C^H_{ij}$ from Refs. [26, 63], but $C_{ij}$ (and consequently $\delta C_{ij}$) are not known at $\mathcal {O}(\alpha _s^3)$. We will argue that using Eq. (13) to define an approximate $C_{ij}$ at N$^3$LO by simply setting to zero the unknown $\mathcal {O}(\alpha _s^3)$ contribution to $\delta C_{ij}$ provides an excellent approximation.

To prove the quality of the approximation, we first observe that if the $\delta C_{ij}$ were unknown the soft part of the pseudo-scalar coefficients would be predicted exactly by the rescaling in Eq. (13). This observation derives from the fact that in Eq. (8) the Sudakov exponential $\exp \mathcal S$ is identical for scalar and pseudo-scalar production, and only $g_0$ contains the process-dependent part. (This, in turn, also shows that the ratio $g_0/g_0^H$ is identical to the ratio of the SCET hard functions *H*’s for the two processes.) Therefore, the approximation based on Eq. (13) is at least as good as a soft-virtual approximation, as it contains the same information. In fact, Eq. (13) contains much more information, thanks to the similarity of the two processes. To see this, we inspect the $\delta C_{ij}$ terms order by order. Defining the $\alpha _s$ expansion as

14 $$\begin{aligned} \delta C_{ij}(z,\alpha _s) = \frac{\alpha _s}{\pi } \delta C_{ij}^{(1)} + \left( \frac{\alpha _s}{\pi }\right) ^2 \delta C_{ij}^{(2)} + \left( \frac{\alpha _s}{\pi }\right) ^3 \delta C_{ij}^{(3)} +\cdots \end{aligned}$$

we first note that, at NLO,

15 $$\begin{aligned} \delta C_{ij}^{(1)} = 0, \end{aligned}$$

since the difference between scalar and pseudo-scalar production at this order is a pure virtual term [35], and therefore fully accounted for by the rescaling. Note that this is already highly non-trivial, as by construction $\delta C_{ij}^{(1)}$ has just to be free of soft-virtual contributions; the fact that the only difference between scalar and pseudo-scalar is corrected by the rescaling is a clear consequence of the similarity between the two processes considered. At the NNLO, we find

16 $$\begin{aligned} \delta C_{gg}^{(2)}&= \frac{495-171z+(20z-2)n_f}{12z}(1-z) \nonumber \\&\quad +\frac{36+21z+2zn_f}{2z}\ln z + \frac{2n_f-27}{3}\ln ^2z \nonumber \\ \delta C_{qg}^{(2)}&= \frac{173-27z}{9z}(1-z) +\frac{24+28z}{3z}\ln z - \frac{28}{9}\ln ^2z \nonumber \\ \delta C_{q\bar{q}}^{(2)}&= 16\frac{10+12z-(1+z)n_f}{27z}(1-z) \nonumber \\&\quad +32\frac{3+8z-zn_f}{27z}\ln z + \frac{32}{27}\ln ^2z \nonumber \\ \delta C_{qq}^{(2)}&= 8\frac{37-3z}{27z}(1-z) +16\frac{6+11z}{27z}\ln z - \frac{64}{27}\ln ^2z \nonumber \\ \delta C_{qq'}^{(2)}&= 8\frac{11-z}{9z}(1-z) +16\frac{2+3z}{9z}\ln z - \frac{16}{9}\ln ^2z. \end{aligned}$$

These results are extremely interesting. We first observe that these terms are next-to-next-to-soft, namely they are suppressed by $(1-z)^2$ with respect to the leading soft terms (i.e., they vanish in $z=1$). Moreover, there are no $\ln (1-z)$ terms, which means that those are predicted exactly for any power of $(1-z)$. Then we observe that at small-*z* these expressions are next-to-next-to-leading logarithmic. Finally, we note that the $\delta C_{ij}$ terms do not contain any explicit scale-dependent contribution at this order.

The fact that the simple rescaling Eq. (13) allows the prediction of all next-to-soft contributions is very promising: it shows that the details of the interaction other than those contained in the virtual contributions are not needed to describe the next-to-soft terms. This observation, if persisting at higher orders (as we conjecture^2^), can be an important step towards the resummation of next-to-soft contributions  [62, 66–71]. Note that the fact that this is true also for the *qg* channel is rather informative, as it tells that the large-*z* logarithms in this channel, which are formally next-to-soft, are encoded in the *gg* subgraphs, as they can be predicted by the knowledge of the virtual *gg* terms.

**Fig. 1:**
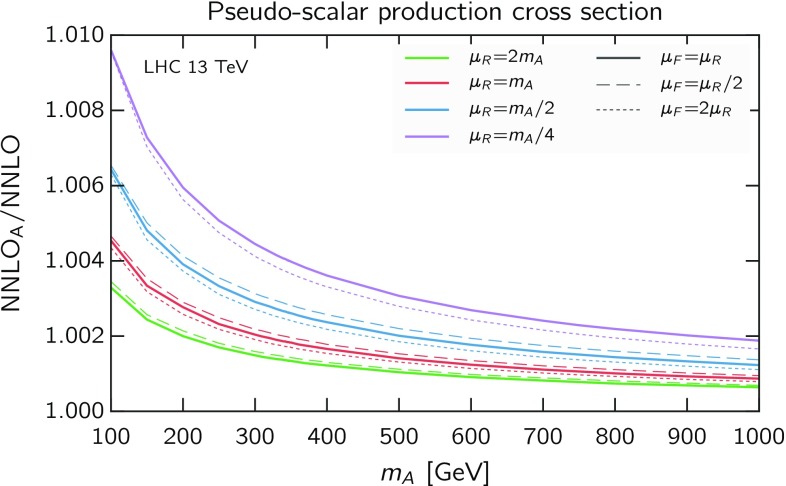
Ratio of approximate NNLO$_\mathrm{A}$ over exact NNLO pseudo-scalar cross sections, as a function of the pseudo-scalar mass $m_A$ at LHC 13 TeV. Curves are shown for four values of $\mu _{\scriptscriptstyle \mathrm R}= 2m_A,m_A,m_A/2,m_A/4$ (*green*, *red*, *blue*, *purple*) and three values of $\mu _{\scriptscriptstyle \mathrm F}/\mu _{\scriptscriptstyle \mathrm R}= 2,1,1/2$ (*dotted*, *solid*, *dashed*)

The other main observation is related to the small-*z* behaviour. In the large-$m_t$ effective theory, the leading small-*z* terms at order $\alpha _s^k$ are of the form $(1/z)\ln ^{2k-1}z$, which were shown to coincide between scalar and pseudo-scalar production processes to all orders in $\alpha _s$ in Ref. [72]. The absence of next-to-leading logarithmic terms of the form $(1/z)\ln ^2z$ in Eq. (16) implies that, at this order, small-*z* contributions in the scalar and pseudo-scalar cases start to differ at the next-to-next-to-leading logarithmic level. This is perhaps not surprising, as in the effective theory the two largest power of the small-*z* logarithms, being double logarithms, are determined by just the hard gluon radiation of the external initial gluon legs. Hence, we expect this to hold at higher orders as well, thus extending the observation of Ref. [72] to the next-to-leading logarithmic terms.

Therefore, we have found that the rescaling Eq. (13), even if the $\delta C_{ij}$ terms are neglected, reproduces exactly the NLO and deviates from the NNLO by terms which are both next-to-next-to-soft and next-to-next-to-leading small-*z*, and thus expected to be small.^3^ To verify this, we plot in Fig. 1 the ratio of the approximate NNLO cross section (denoted NNLO$_\mathrm{A}$, as obtained setting $\delta C_{ij}=0$) over the exact one, for a range of pseudo-scalar masses and for various choices of the scales. (The setting of PDFs and other parameters is the same as in Sect. 5.) At high masses, i.e. closer to threshold, the difference is at most $\sim 2$‰, depending on the value of the renormalisation scale, but almost independent of the factorisation scale (a consequence of the fact that the factorisation scale dependence is generally mild for this process). At smaller masses, where unpredicted next-to-next-to-soft corrections are larger, the discrepancy can reach $\sim$1% for small renormalisation scales. Overall, the agreement is excellent.

At the next order, we do not have the exact result and therefore we cannot compare. However, we expect that the $\delta C_{ij}^{(3)}$ coefficients share the same features of the $\delta C_{ij}^{(2)}$, and as such their contribution should be very small, also considering that the N$^3$LO correction itself is much smaller than the NNLO one. Numerically, based on the NNLO comparison, we expect the difference of our approximate N$^3$LO$_\mathrm{A}$ result to the exact to be just a few permille, and therefore smaller than scale variation and many other sources of uncertainties. To further support this expectation, we consider “variations” of the approximation itself to probe the effects of the unknown contributions at N$^3$LO. The third-order coefficient $C_{ij}^{(3)}$ is given, according to Eq. (13), and using explicitly Eq. (15), by

17 $$\begin{aligned} C_{ij}^{(3)}(z)&= C_{ij}^{H(3)}(z) + r^{(1)} C_{ij}^{H(2)}(z) + r^{(2)} C_{ij}^{H(1)}(z) \nonumber \\&\quad + r^{(3)} C_{ij}^{H(0)}(z) + \delta C_{ij}^{(3)}(z) + r^{(1)}\delta C_{ij}^{(2)}(z), \end{aligned}$$

where $C_{ij}^{H(k)}(z)$ are the expansion coefficients of $C_{ij}^H(z,\alpha _s)$ and $r^{(k)}$ are the expansion coefficients of the ratio $g_0(\alpha _s)/g_0^H(\alpha _s)$. Our N$^3$LO$_\mathrm{A}$ is defined by dropping the $\delta C_{ij}^{(3)}(z)$ term in Eq. (17). We could equally decide to also drop the last term in the equation, which would be natural if we had defined $\delta C_{ij}$ differently, with the rescaling in Eq. (13) applied only to $C_{ij}^H$ and not to $\delta C_{ij}$. With this modified definition we obtain a N$^3$LO prediction which only differs by less than 0.3‰ from the N$^3$LO$_\mathrm{A}$ in the considered range of masses and scales (same as Fig. 1). This excellent agreement might not be too significant, as it derives from the $\delta C_{ij}^{(2)}(z)$ term, and can therefore be expected to be roughly the same effect seen at NNLO suppressed by the factor $\alpha _sr^{(1)}\sim 0.03$, so in particular it does not take into account possible larger corrections in the unknown $\delta C_{ij}^{(3)}(z)$ contribution.

**Fig. 2:**
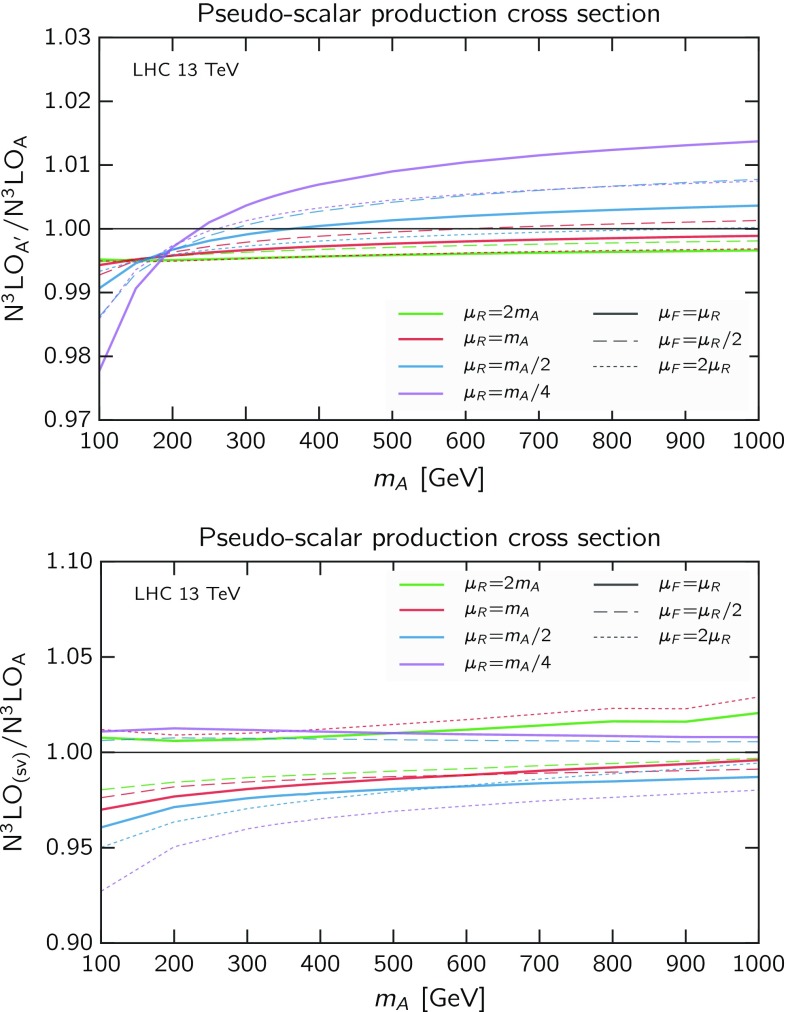
Ratio over approximate N$^3$LO$_\mathrm{A}$ of the variant approximate N$^3$LO$_\mathrm{A'}$ described in the text (*upper panel*) and of the soft-virtual N$^3$LO$_\mathrm{(sv)}$ (*lower panel*). Curves are as in Fig. 1

Alternatively, and more drastically, we could ignore the rescaling and drop all the terms in Eq. (17) except the first: in this case, the ignored terms contain also leading soft and next-to-soft contributions. Hence, this variation provides a conservative estimate of the error on the approximation, as it also varies contributions (the soft-virtual ones) which are known and correctly included in our N$^3$LO$_\mathrm{A}$. This variation is also useful to understand how big corrections can be if our conjecture on the form of $\delta C_{ij}$, namely the absence of next-to-soft terms in them, was wrong. The ratio of this alternative approximation (denoted in the plot N$^3$LO$_\mathrm{A'}$) over our default N$^3$LO$_\mathrm{A}$ is shown in Fig. 2 (upper panel), where it clearly appears that the largest variation never exceeds $2\%$, and is smaller than $1\%$ for most scales and masses.

Based on these considerations, we would conclude that a realistic uncertainty on our approximate result is of the order of $1\%$. In addition, one should also consider the uncertainty coming from the fact that the scalar Higgs N$^3$LO cross section is itself not known exactly, but as a threshold expansion up to order $(1-z)^{37}$ [26, 63]. The uncertainty coming from the truncation of the threshold expansion has been estimated to be $0.37\%$ for the SM Higgs boson at the 13 TeV LHC [26]. Since the relative size of the perturbative contributions at various orders is roughly the same for scalar and pseudo-scalar, this value applies also to our case, for the same mass. At higher masses the process gets closer to threshold, and the threshold expansion converges more rapidly and is less contaminated by small-*z* terms (which are not predicted correctly in the threshold expansion), so the uncertainty from the truncation is likely smaller. Therefore, the final estimate on the uncertainty on our result remains at the percent level.

**Fig. 3:**
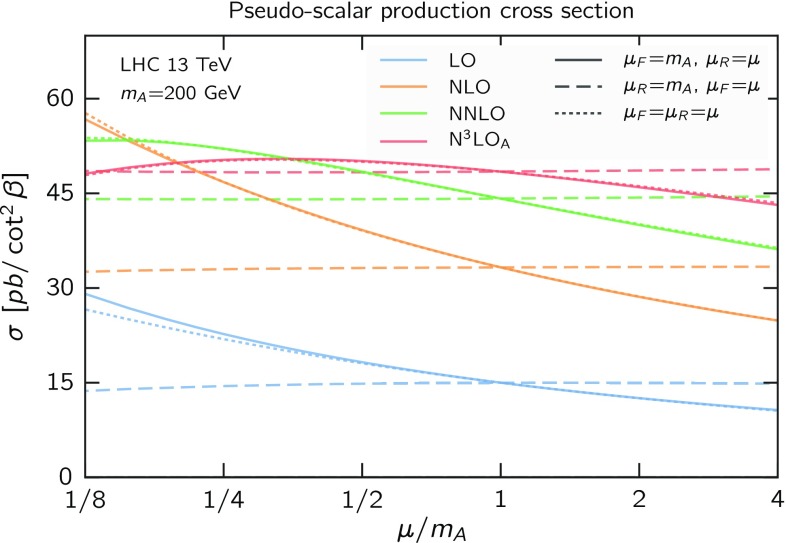
Renormalisation (*solid*), factorisation (*dashed*) and simultaneous (*dotted*) scale dependence for pseudo-scalar production with $m_A=200$ GeV at LHC 13 TeV. Results at LO (*blue*), NLO (*orange*), NNLO (*green*) and N$^3$LO$_\mathrm{A}$ (*red*) are shown

We now consider the *modified* soft-virtual (SV) approximation proposed in Ref. [30], denoted as N$^3$LO$_\mathrm{(sv)}$. It consists in approximating the third-order coefficient function by the threshold plus-distributions multiplied by an overall factor *z*. This approximation proved to be more powerful at previous orders, and it works better in the case of the SM Higgs. The reason can be traced back to the fact that this modified version includes some collinear contributions, as proposed in Ref. [61]. Indeed, we notice that this modified SV approximation is close in spirit to the soft$_1$ approximation of Ref. [61], where $\ln z$ contributions were also retained. In Fig. 2 (lower panel) we plot the ratio of this N$^3$LO$_\mathrm{(sv)}$ prediction over our N$^3$LO$_\mathrm{A}$ result, for several choices of scales. The agreement is typically within 5%, improving down to 2–3% at large masses, when the process is closer to threshold and the soft-virtual approximation is more accurate. We also not that the $\mu _{\scriptscriptstyle \mathrm F}$ dependence is significant, since in the N$^3$LO$_\mathrm{(sv)}$ it is included only in the *gg* channel, and is therefore unbalanced.

To conclude this section, we present the dependence upon renormalisation (solid), factorisation (dashed) and simultaneous (dotted) scale variation in Fig. 3 for LO (blue), NLO (orange), NNLO (green) and N$^3$LO$_\mathrm{A}$ (red). We consider a pseudo-scalar mass $m_A=200$ GeV at LHC with $\sqrt{s}=13$ TeV. While $\mu _{\scriptscriptstyle \mathrm F}$ dependence is very flat even at low orders, $\mu _{\scriptscriptstyle \mathrm R}$ dependence flattens out significantly at N$^3$LO$_\mathrm{A}$. Simultaneous variation of $\mu _{\scriptscriptstyle \mathrm R}$ and $\mu _{\scriptscriptstyle \mathrm F}$ is very similar to $\mu _{\scriptscriptstyle \mathrm R}$ variation. These results are very similar to those for scalar Higgs production [26].

**Fig. 4:**
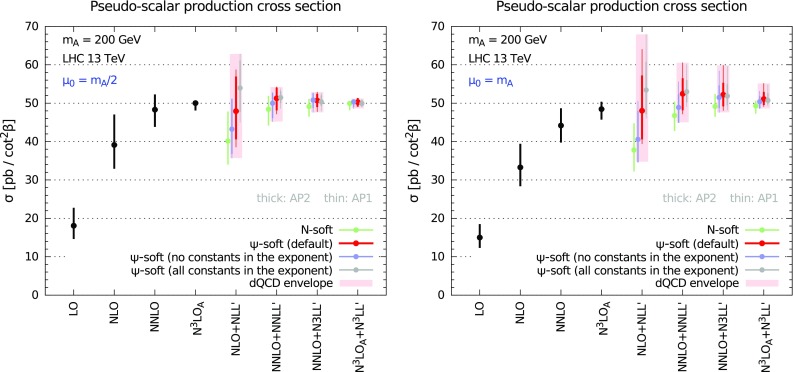
Fixed-order (*black*) and resummed cross section in dQCD at various orders for $m_A=200$ GeV at the 13 TeV LHC. The standard *N*-soft resummation is shown together with the various variants of $\psi$-soft resummation as discussed in the text. The envelope of the various $\psi$-soft resummed results is shown as *light-red* rectangles

## Numerical results at N$^3$LO$_\text {A}$+N$^3$LL$^\prime$

We now present the results for the inclusive pseudo-scalar cross section in gluon–gluon fusion at N$^3$LO$_\mathrm{A}$+N$^3$LL$^\prime$ accuracy at LHC $\sqrt{s}=13$ TeV. We use the NNLO set of parton distributions NNPDF30_nnlo_as_0118 [74] with $\alpha _s= 0.118$ through the LHAPDF 6 interface [75]. In this study we assume that the pseudo-scalar couples only to top quark and we take $m_{t}=173.2$ GeV. We have implemented the exact NNLO and the approximate N$^3$LO$_\mathrm{A}$ results for pseudo-scalar production in the public code ggHiggs [28, 61, 76, 77]. We then use the public code TROLL [27, 29, 78] to perform the resummation in the dQCD and SCET formalism.

We recall that there have been a series of experimental searches at the LHC for a pseudo-scalar boson in gluon fusion as well as bottom associated production channels. For instance, the ATLAS collaboration has searched for pseudo-scalar boson over the mass window $200\; \text {GeV}< m_A< 1200\; \text {GeV}$ using 13 TeV data and has put 95% confidence level (CL) upper limits on the production cross section times the branching fraction as well as 95% CL exclusion limits on the model parameter $\tan \beta$ as a function of $m_A$ in different supersymmetric scenarios. For example, with data corresponding to a luminosity of 3.2 fb$^{-1}$ [79], the excluded region is $\tan \beta > 7 (47)$ for $m_A= 200 (1000)$ GeV while with luminosity of 13.3 fb$^{-1}$ [80] the excluded region is $\tan \beta > 9 (42)$ for $m_A= 200 (1200)$ GeV in hMSSM scenarios [81]. Therefore, at the moment, no mass value is excluded, provided the model parameter $\tan \beta$ is in the allowed range.

We first focus on an hypothetical pseudo-scalar mass $m_A=200$ GeV. In Fig. 4 we show the inclusive cross section at fixed LO, NLO, NNLO and N$^3$LO$_\mathrm{A}$ accuracy, and at NLO+NLL$^\prime$, NNLO+NNLL$^\prime$, NNLO+N$^3$LL$^\prime$, and N$^3$LO$_\mathrm{A}$+N$^3$LL$^\prime$ accuracy in the dQCD approach. We consider two different values for the central factorisation and renormalisation scale $\mu _{\scriptscriptstyle \mathrm F}=\mu _{\scriptscriptstyle \mathrm R}=\mu _0$, namely $\mu _0=m_A/2$ (left panel) and $\mu _0=m_A$ (right panel).

We included in our results both the NNLO+N$^3$LL$^\prime$ and the N$^3$LO$_\mathrm{A}$+N$^3$LL$^\prime$ cross sections. These two constructions have the same fixed order up to $\mathcal {O}(\alpha _s^2)$, and share the same all-order resummed contributions from $\mathcal {O}(\alpha _s^4)$ onwards. However, the contribution of $\mathcal {O}(\alpha _s^3)$ is different in the two results: in the N$^3$LO$_\mathrm{A}$+N$^3$LL$^\prime$ it is given by our approximation of Sect. 4, while in the NNLO+N$^3$LL$^\prime$ it is given by the N$^3$LL$^\prime$ resummation expanded to $\mathcal {O}(\alpha _s^3)$. In other words, in absence of a full N$^3$LO computation, both provide alternative ways of estimating the N$^3$LO, which share the same soft, virtual and collinear contributions. Since in our results we vary the resummation prescription, the NNLO+N$^3$LL$^\prime$ also contains an estimate of the uncertainty on the N$^3$LO itself, and therefore can be considered as a (much) more conservative estimate of the unknown exact N$^3$LO+N$^3$LL$^\prime$ cross section.

We show the results obtained using different resummation prescriptions. Following the approach of Ref [28], predictions are shown for the *N*-soft and for variants of the $\psi$-soft prescriptions which differ by subleading and/or subdominant contributions, as discussed in Sect. 3. For each variant we perform a 7-point scale variation varying $\mu _{\scriptscriptstyle \mathrm F}$ and $\mu _{\scriptscriptstyle \mathrm R}$ by a factor 2 up or down and keeping $1/2 \le \mu _{\scriptscriptstyle \mathrm R}/\mu _{\scriptscriptstyle \mathrm F}\le 2$. The final uncertainty on our predictions is computed as the envelope of the different $\psi$-soft variants and each scale variation, and it is shown as light-red rectangles in Fig. 4. The uncertainty of the fixed-order results is computed as a canonical 7-point scale variation.

**Fig. 5:**
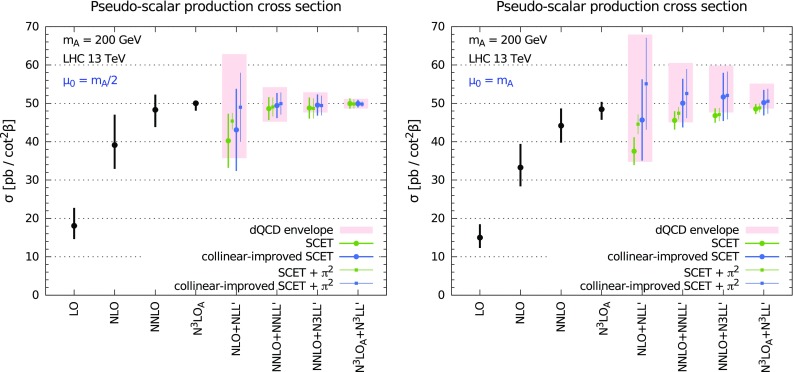
Same as Fig. 4 but showing the SCET resummed results. The dQCD envelope is also shown to facilitate the comparison

**Fig. 6:**
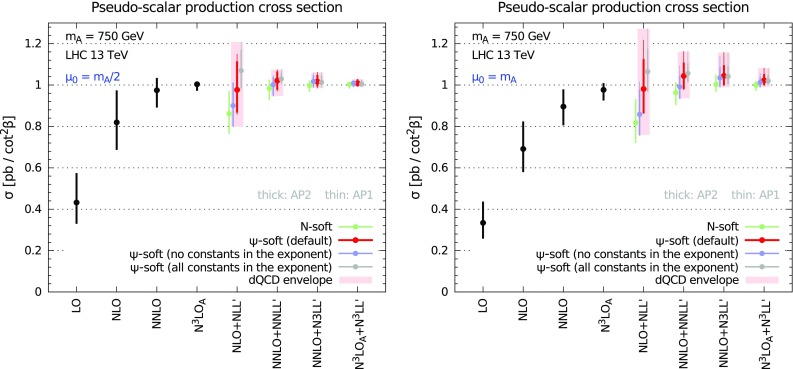
Same as Fig. 4 but for $m_A=750$ GeV

As for the SM Higgs, the fixed-order perturbative expansion displays poor convergence, especially at lower orders. In particular, the NLO correction is more than $100\%$ larger than the LO, and the NNLO is a significant correction over the NLO. Ignoring the LO, which does not contain enough information, we can focus on the behaviour of the series at higher orders. Because of the large perturbative corrections, canonical scale variation does not guarantee a reliable estimate of the uncertainty from missing higher orders. In particular, the NNLO central value is not contained in the NLO uncertainty band, and the NNLO and the NLO uncertainty bands do not even overlap at $\mu _0=m_A$. The N$^3$LO$_\mathrm{A}$ is a smaller correction, perhaps an indication that the series is finally converging. The N$^3$LO$_\mathrm{A}$ value is contained in the NNLO uncertainty band, yet is not contained in the NLO uncertainty band; again there is no overlap of the two bands.

Nonetheless, a robust estimate of the missing higher-order uncertainty can be attained by resorting to resummation. On one hand, resummed results exhibit a better perturbative behaviour, thereby suggesting that convergence is improved when resummed contributions are included. On the other hand, variation of subleading and subdominant contributions on top of scale variation provides a more robust method for estimating higher-order uncertainty. Contrarily to the fixed order, the NLO+NLL$^\prime$ total band fully envelops the NNLO+NNLL$^\prime$ band, and the NNLO+N$^3$LL$^\prime$ and N$^3$LO$_\mathrm{A}$+N$^3$LL$^\prime$ are contained in the NNLO+NNLL$^\prime$ band, which also cover the central value of the N$^3$LO$_\mathrm{A}$ result. A similar pattern is observed also if only the default $\psi$-soft prescription is considered. This confirms the conclusions of Ref. [28] in the context of SM Higgs production and extends them to the case of pseudo-scalar Higgs production. Similarly, we also confirm that the central scale choice $\mu _0=m_A/2$ seems a better one, as it leads to faster convergence and smaller, yet reliable, final uncertainty.

We now analyse the impact of resummation in a framework complementary to the dQCD approach. In Fig. 5 we compare the fixed-order results with variants of the resummed results obtained in the SCET formalism. We perform two different choices of the soft logarithms and we consider the effect of the $\pi ^2$ resummation, as discussed in Sect. 3. For each of the variants we compute the uncertainty as in Ref. [44]. Specifically, we vary independently $\mu _{\scriptscriptstyle \mathrm F}$, $\mu _{\scriptscriptstyle \mathrm H}$ and $\mu _{\scriptscriptstyle \mathrm S}$, keeping the other scales fixed when one is varied. As far as $\mu _{\scriptscriptstyle \mathrm F}$ and $\mu _{\scriptscriptstyle \mathrm H}$ are concerned, they are varied by a factor of two up and down, about the central scale $\mu _0$, which we again take to be either $\mu _0=m_A/2$ (left panel) or $\mu _0=m_A$ (right panel). The definition of the central $\mu _{\scriptscriptstyle \mathrm S}$ and of its variation range is more complicated, and we refer to Ref. [44] for a detailed explanation. For each scale, the largest variation is then symmetrised, and the final (symmetric) uncertainty is obtained by adding each individual uncertainty in quadrature. To facilitate the comparison with the dQCD results, in Fig. 5 we also show the envelope of the $\psi$-soft variants in dQCD as light-red rectangles.

We observe that the original formulation of SCET resummation of Ref. [44] leads to a small correction of the fixed-order result, due to the choice of the soft logarithms. Furthermore, the uncertainty bands are comparable or smaller than their fixed-order counterparts, suggesting an underestimate of the theory errors. On the contrary, the impact of resummation is more significant if subleading terms are included in the form of the collinear improvement of Ref. [29]. In this collinear-improved variant the bands are larger and always overlap, indicating a better perturbative stability. The inclusion of $\pi ^2$ resummation further speeds up the convergence at $\mu _0=m_A/2$. The spread of the variants we have considered lies almost entirely in the dQCD envelope, with the exception of the NNLO+N$^3$LL$^\prime$ and N$^3$LO$_\mathrm{A}$+N$^3$LL$^\prime$ without collinear improvement in the $\mu _0=m_A$ case.^4^ Finally, we observe that the central scale $\mu _0=m_A/2$ turns out to be a better choice also from the point of view of SCET resummation, both because the errors are smaller, and because the impact of higher orders is reduced, as one can understand from the smaller difference between the original and the collinear-improved versions.

In Fig. 6 we show the dQCD predictions for a larger pseudo-scalar mass, $m_A=750$ GeV. This mass value was of some interest in the light of recent measurements [82, 83]. We observe exactly the same pattern found for $m_A=200$ GeV. The only important difference is that the final uncertainty on the resummed results at $\mu _0=m_A/2$ is smaller than for the lower mass value, probably due to the fact that at larger masses the process is closer to threshold and the resummation is therefore more accurate (i.e. less uncertain) in describing the higher orders. We do not show the analogous results for SCET resummation, as they have the same features of the lower mass results. We then conclude that all the observations made for $m_A=200$ GeV remain unchanged for any pseudo-scalar mass.

The comparison of the SCET results with the dQCD ones confirms the procedure suggested in Ref. [28] as a robust and reliable method for computing the uncertainty from missing higher orders, and confirms the scale $\mu _0=m_A/2$ as an optimal central scale. We can now therefore use this procedure to provide precise and accurate predictions for pseudo-scalar production at the LHC for generic values of the pseudo-scalar mass $m_A$.

**Table 1:** Resummed cross section at N$^3$LO$_\mathrm{A}$+N$^3$LL$^\prime$ accuracy in dQCD for different values of $m_A$ at the 13 TeV LHC. The density of $m_A$ values increases close to the $t\bar{t}$ threshold to accurately describe the peak. The error corresponds to the dQCD envelope

$m_A$[GeV]	$\sigma _{{\text {N}}^3{\text {LO}}_\mathrm{A}+{\text {N}}^3{\text {LL}}^\prime }$[pb/$\cot ^2\beta$]
100	$1.71_{-0.08}^{+0.06}\times 10^{+2}$
150	$8.29_{-0.32}^{+0.25}\times 10^{+1}$
200	$5.03_{-0.16}^{+0.09}\times 10^{+1}$
250	$3.64_{-0.10}^{+0.07}\times 10^{+1}$
300	$3.22_{-0.09}^{+0.06}\times 10^{+1}$
310	$3.27_{-0.08}^{+0.06}\times 10^{+1}$
320	$3.39_{-0.09}^{+0.06}\times 10^{+1}$
330	$3.66_{-0.09}^{+0.07}\times 10^{+1}$
340	$4.28_{-0.11}^{+0.08}\times 10^{+1}$
341	$4.39_{-0.11}^{+0.08}\times 10^{+1}$
342	$4.53_{-0.11}^{+0.08}\times 10^{+1}$
343	$4.69_{-0.12}^{+0.09}\times 10^{+1}$
344	$4.90_{-0.12}^{+0.09}\times 10^{+1}$
345	$5.18_{-0.13}^{+0.09}\times 10^{+1}$
346	$5.68_{-0.14}^{+0.10}\times 10^{+1}$
347	$6.33_{-0.16}^{+0.12}\times 10^{+1}$
348	$6.24_{-0.15}^{+0.11}\times 10^{+1}$
349	$6.16_{-0.15}^{+0.11}\times 10^{+1}$
350	$6.07_{-0.15}^{+0.11}\times 10^{+1}$
360	$5.28_{-0.13}^{+0.10}\times 10^{+1}$
370	$4.60_{-0.11}^{+0.08}\times 10^{+1}$
380	$4.02_{-0.10}^{+0.07}\times 10^{+1}$
390	$3.53_{-0.08}^{+0.06}\times 10^{+1}$
400	$3.10_{-0.07}^{+0.06}\times 10^{+1}$
500	$9.71_{-0.20}^{+0.18}\times 10^{+0}$
600	$3.60_{-0.07}^{+0.07}\times 10^{+0}$
700	$1.51_{-0.03}^{+0.03}\times 10^{+0}$
750	$1.01_{-0.02}^{+0.02}\times 10^{+0}$
800	$6.89_{-0.11}^{+0.14}\times 10^{-1}$
900	$3.38_{-0.05}^{+0.07}\times 10^{-1}$
1000	$1.75_{-0.03}^{+0.04}\times 10^{-1}$

**Fig. 7:**
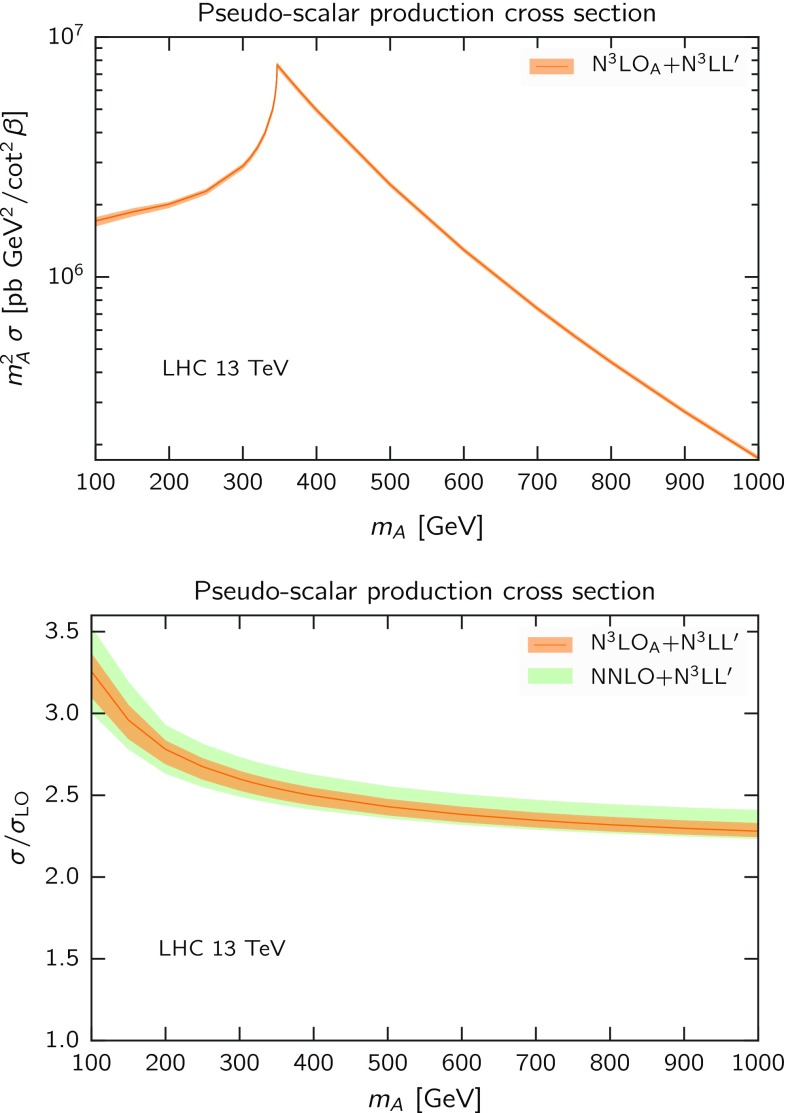
Resummed cross section at N$^3$LO$_\mathrm{A}$+N$^3$LL$^\prime$ accuracy in dQCD as a function of $m_A$ at the 13 TeV LHC. We show both the absolute cross section (*upper panel*), multiplied by $m_A^2$ for readability, and the *K*-factor $\sigma _{\mathrm{{N}^3}\mathrm{LO}_\mathrm{{A}}+\mathrm{{N}}^3\mathrm{{LL}}^\prime } /\sigma _{\text {LO}}$ (*lower panel*). In the *lower panel* we also include the prediction for NNLO+N$^3$LL$^\prime$. The error shown corresponds to the dQCD envelope

In Table 1 we collect the predictions for the inclusive cross section for pseudo-scalar production at LHC 13 TeV for different values of $m_A$ between 100 GeV and 1 TeV. For each mass value we show predictions at N$^3$LO$_\mathrm{A}$+N$^3$LL$^\prime$. The central value of the resummed result is computed using the default variant of $\psi$-soft$_2$ and the uncertainty is computed as previously discussed, i.e. as the envelope of the different $\psi$-soft variants computed for each scale variation about the central scale $\mu _{\scriptscriptstyle \mathrm R}=\mu _{\scriptscriptstyle \mathrm F}=m_A/2$. The predictions at N$^3$LO$_\mathrm{A}$+N$^3$LL$^\prime$ are also collected in the form of a plot in Fig. 7, where we show the resummed cross section and the *K*-factor $\sigma _{\text {N}^3\mathrm{LO}_\mathrm{A}+\mathrm{{N}}^3\mathrm{LL}^\prime }/\sigma _{\text {LO}}$ as a function of $m_A$ (orange curve and band). In the *K*-factor plot we also show, in green, the NNLO+N$^3$LL$^\prime$ uncertainty band. It is apparent that the knowledge of the N$^3$LO improves significantly the precision of the prediction, as the error band of the N$^3$LO$_\mathrm{A}$+N$^3$LL$^\prime$ result is approximately half of the NNLO+N$^3$LL$^\prime$ band. The latter can be interpreted as a more conservative uncertainty, covering the uncertainty on the approximate N$^3$LO$_\mathrm{A}$ result as estimated in Sect. 4.

Note that the large-$m_t$ assumption of the effective field theory approach used here is violated for pseudo-scalar masses $m_A\gtrsim 2m_t$, namely close and after the peak in the upper panel of Fig. 7. However, it is well known (e.g. [36, 84, 85]) that the effective theory approach, when rescaled with the exact LO result, Eq. (3), provides a reasonably good approximation even at large masses, outside the region of formal validity of the effective approach. This can be understood in terms of the dominance of soft-collinear contributions, which indeed factorise. Indeed, the difference between the exact and the effective theory results at NLO reaches $\sim 10\%$ for $m_A\gtrsim 500$ GeV [86, 87], but does not increase much as $m_A$ gets larger. The residual effect from missing NNLO finite-$m_t$ terms can then be expected to be a few percent, as it happens in the scalar case [88, 89]. Therefore, once the known NLO finite-$m_t$ corrections are included, our results are expected to be reasonably accurate for phenomenology.

## Conclusions

In this work we presented precise predictions for pseudo-scalar Higgs boson production at LHC based on a combination of fixed order at exact NNLO plus approximate N$^3$LO and of threshold resummation at N$^3$LL$^\prime$.

We have proposed a new method for predicting the N$^3$LO cross section, based on the similarity of the pseudo-scalar production process in gluon fusion with the analogous scalar production process, for which the exact N$^3$LO result has been recently made available. This method consists in a simple rescaling of the perturbative scalar coefficient functions by the ratio of the process-dependent functions $g_0$ (or hard functions *H*) of the resummation for the two processes. By construction, this procedure reproduces exactly the soft-virtual-collinear contributions of the pseudo-scalar coefficients. Interestingly, up to NNLO where the exact result is known, this procedure also reproduces all next-to-soft terms, all next-to-leading small-*z* logarithms and all the terms proportional to $\ln (1-z)$ to any positive power. Assuming this pattern remains true at N$^3$LO and beyond, these observations can also give some insight on the structure and origin of next-to-soft contributions and how to perform their all-order resummation. In this work, this allowed us to construct a precise approximation to the N$^3$LO cross section, up to corrections estimated to be at the percent level.

We then studied the effect of including threshold resummation at N$^3$LL$^\prime$. We considered threshold resummation both in the traditional direct QCD approach and in the effective SCET approach. We pay particular attention to the effect of including subleading logarithmic and subleading power (i.e., beyond threshold) contributions in the resummations. Following Ref. [28], we vary these subleading contributions in dQCD to obtain a rather conservative uncertainty estimate due to missing higher orders. This estimate, computed as the envelope of scale and subleading-term variations of the resummed result, is very reliable, as demonstrated by the fact that the resulting error band successfully covers the next orders. Specifically, it is much more reliable than the uncertainty estimated by scale variation at fixed order, which typically underestimates the size of higher-order contributions. Comparison to SCET results further validates the reliability of the dQCD approach.

Differently from the fixed-order results, the resummed results are very stable upon variation of the central scale, except for the size of the error band which is somewhat dependent on it. We identify $\mu _{\scriptscriptstyle \mathrm R}=\mu _{\scriptscriptstyle \mathrm F}=m_A/2$ as an optimal central scale, in the sense that the dQCD error band turns out to be rather small, but still reliable as demonstrated by the previous orders and the comparison with SCET. We therefore use this choice to present resummed pseudo-scalar production cross sections for a wide range of pseudo-scalar masses, from $m_A=100$ GeV to $m_A=1$ TeV. The *K*-factor with respect to the LO cross section ranges from $\sim 3.3$ to $\sim 2.3$, respectively, and the uncertainty estimate from missing higher-order ranges from approximately $\pm 4\%$ at small mass to approximately $\pm 2\%$ at high mass. We observe, however, that finite top-quark mass effects, neglected in our large-$m_t$ effective theory approach, become sizeable at large $m_A$. After including the known NLO corrections, the residual effect from missing NNLO finite $m_t$ contributions can possibly reach a few percent for $m_A\gtrsim 2m_t$.

Our results, although obtained assuming a Two Higgs Doublet Model like the MSSM for pseudo-scalar boson interactions, can be trivially extended to other more exotic models by simply changing the Wilson coefficient of the large-$m_t$ effective theory, which encodes the full-theory information. The approximate N$^3$LO$_\mathrm{A}$ is available through the public code ggHiggs [77], v3.3 onwards, and the threshold resummation up to N$^3$LL$^\prime$ is available in the public code TROLL [78], v3.1 onwards.

## Footnotes

1 Here the prime $^\prime$ means that in addition to the N$^3$LL terms the resummed result includes additional (formally higher logarithmic order) terms coming from the matching to N$^3$LO. For a more detailed discussion, see Refs. [27, 29]. We stress that in most of the literature the prime is omitted.

2 To support our conjecture, we have tested Eq. (13) on two $q\bar{q}$ dominated processes: Drell–Yan and $b\bar{b}H$ production. Also in this case, we find that the $\delta C_{ij}$ coefficients are next-to-next-to-soft, even though they are non-zero already at NLO, and terms proportional to powers of $\log (1-z)$ appear at NNLO.

3 We observe that for scalar Higgs production an approximation based on soft and next-to-soft terms only is in principle not sufficient to determine the full cross section at high accuracy, neither at N$^3$LO [26, 63] and not even at NLO and NNLO [73]. This derives from the fact that terms at next-to-next-to-soft and beyond are not uniquely defined, and can be (somewhat artificially) modified at will by a proper redefinition of the expansion parameters, leading to sizeable effects. Here, differently, the definition of $\delta C_{ij}$ is unique, and the size of their contribution can only be assessed by a direct evaluation, which (as we shall see) gives a small effect. This can be understood by the fact that the rescaling Eq. (13) also predicts some (but not all) next-to-next-to-soft terms and higher, and in particular those coming from the $P_{gg}$ splitting function associated with soft radiation, which are universal and drive most of the higher soft-order corrections [61].

4 This is not surprising, since it is known [29, 61] that the choice of logarithms performed in [44] underestimates the full result if expanded in powers of $\alpha _s$. Anyway, the difference is not dramatic, and had one symmetrised the dQCD envelope about the central $\psi$-soft prediction they would be contained in the band.

## References

[1] ATLAS Collaboration, G. Aad et al., Observation of a new particle in the search for the Standard Model Higgs boson with the ATLAS detector at the LHC. Phys. Lett. B**716**, 1–29 (2012). arXiv:1207.7214

[2] Collaboration CMS, Chatrchyan S (2012). Observation of a new boson at a mass of 125 GeV with the CMS experiment at the LHC. Phys. Lett. B.

[3] Higgs PW (1964). Broken symmetries, massless particles and gauge fields. Phys. Lett..

[4] Higgs PW (1964). Broken symmetries and the masses of gauge bosons. Phys. Rev. Lett..

[5] Higgs PW (1966). Spontaneous symmetry breakdown without massless bosons. Phys. Rev..

[6] Englert F, Brout R (1964). Broken symmetry and the mass of gauge vector mesons. Phys. Rev. Lett..

[7] Guralnik GS, Hagen CR, Kibble TWB (1964). Global conservation laws and massless particles. Phys. Rev. Lett..

[8] ATLAS Collaboration, Combined coupling measurements of the Higgs-like boson with the ATLAS detector using up to 25 fb$$^{-1}$$ of proton-proton collision data

[9] CMS Collaboration, V. Khachatryan et al., Constraints on the spin-parity and anomalous HVV couplings of the Higgs boson in proton collisions at 7 and 8 TeV. Phys. Rev. D **92**(1), 012004 (2015). arXiv:1411.3441

[10] ATLAS Collaboration, G. Aad et al., Evidence for the spin-0 nature of the Higgs boson using ATLAS data. Phys. Lett. B **726**, 120–144 (2013). arXiv:1307.1432

[11] Fayet P (1975). Supergauge invariant extension of the Higgs mechanism and a model for the electron and its neutrino. Nucl. Phys. B.

[12] Fayet P (1976). Supersymmetry and weak, electromagnetic and strong interactions. Phys. Lett. B.

[13] Fayet P (1977). Spontaneously broken supersymmetric theories of weak, electromagnetic and strong interactions. Phys. Lett. B.

[14] Dimopoulos S, Georgi H (1981). Softly broken supersymmetry and SU(5). Nucl. Phys. B.

[15] Sakai N (1981). Naturalness in supersymmetric guts. Z. Phys. C.

[16] K. Inoue, A. Kakuto, H. Komatsu, S. Takeshita, Aspects of grand unified models with softly broken supersymmetry, Prog. Theor. Phys.**68**, 927 (1982). [Erratum: Prog. Theor. Phys. 70, 330 (1983)]

[17] Inoue K, Kakuto A, Komatsu H, Takeshita S (1984). Renormalization of supersymmetry breaking parameters revisited. Prog. Theor. Phys..

[18] Inoue K, Kakuto A, Komatsu H, Takeshita S (1982). Low-energy parameters and particle masses in a supersymmetric grand unified model. Prog. Theor. Phys..

[19] S.P. Martin, Three-loop corrections to the lightest Higgs scalar boson mass in supersymmetry. Phys. Rev. D **75**, 055005 (2007). arXiv:hep-ph/0701051

[20] R.V. Harlander, P. Kant, L. Mihaila, M. Steinhauser, Higgs boson mass in supersymmetry to three loops. Phys. Rev. Lett. **100**, 191602 (2008). arXiv:0803.0672. [Phys. Rev. Lett. 101, 039901 (2008)] 10.1103/PhysRevLett.100.191602 18518437

[21] Kant P, Harlander RV, Mihaila L, Steinhauser M (2010). Light MSSM Higgs boson mass to three-loop accuracy. JHEP.

[22] R.V. Harlander, W.B. Kilgore, Production of a pseudoscalar Higgs boson at hadron colliders at next-to-next-to leading order. JHEP **10**, 017 (2002). arXiv:hep-ph/0208096 10.1103/PhysRevLett.88.201801 12005555

[23] Anastasiou C, Melnikov K (2003). Pseudoscalar Higgs boson production at hadron colliders in NNLO QCD. Phys. Rev. D.

[24] V. Ravindran, J. Smith, W.L. van Neerven, NNLO corrections to the total cross-section for Higgs boson production in hadron hadron collisions. Nucl. Phys. B **665**, 325–366 (2003). arXiv:hep-ph/0302135

[25] Anastasiou C, Duhr C, Dulat F, Herzog F, Mistlberger B (2015). Higgs Boson gluon-fusion production in QCD at three loops. Phys. Rev. Lett..

[26] Anastasiou C, Duhr C, Dulat F, Furlan E, Gehrmann T, Herzog F, Lazopoulos A, Mistlberger B (2016). High precision determination of the gluon fusion Higgs boson cross-section at the LHC. JHEP.

[27] Bonvini M, Marzani S (2014). Resummed Higgs cross section at N$$^{3}$$LL. JHEP.

[28] Bonvini M, Marzani S, Muselli C, Rottoli L (2016). On the Higgs cross section at N$$^{3}$$ LO+N$$^{3}$$LL and its uncertainty. JHEP.

[29] M. Bonvini, L. Rottoli, Three loop soft function for N$$^3$$LL$$^\prime $$ gluon fusion Higgs production in soft-collinear effective theory. Phys. Rev. D **91**(5), 051301 (2015). arXiv:1412.3791

[30] T. Ahmed, M.C. Kumar, P. Mathews, N. Rana, V. Ravindran, Pseudo-scalar Higgs boson production at threshold N$$^3$$ LO and N$$^3$$ LL QCD. Eur. Phys. J. C **76**(6), 355 (2016). arXiv:1510.02235 10.1140/epjc/s10052-016-4510-1 PMC5335642 28316498

[31] de Florian D, Zurita J (2008). Soft-gluon resummation for pseudoscalar Higgs boson production at hadron colliders. Phys. Lett. B.

[32] T. Schmidt, M. Spira, Higgs boson production via gluon fusion: soft-gluon resummation including mass effects. Phys. Rev. D **93**(1), 014022 (2016). arXiv:1509.00195

[33] R.P. Kauffman, W. Schaffer, QCD corrections to production of Higgs pseudoscalars. Phys. Rev. D **49**, 551–554 (1994). arXiv:hep-ph/9305279 10.1103/physrevd.49.551 10016794

[34] A. Djouadi, M. Spira, P.M. Zerwas, Two photon decay widths of Higgs particles. Phys. Lett. B **311**, 255–260 (1993). arXiv:hep-ph/9305335

[35] Spira M, Djouadi A, Graudenz D, Zerwas PM (1993). SUSY Higgs production at proton colliders. Phys. Lett. B.

[36] M. Spira, A. Djouadi, D. Graudenz, P.M. Zerwas, Higgs boson production at the LHC. Nucl. Phys. B **453**, 17–82 (1995). arXiv:hep-ph/9504378

[37] Harlander R, Kant P (2005). Higgs production and decay: analytic results at next-to-leading order QCD. JHEP.

[38] Pak A, Rogal M, Steinhauser M (2011). Production of scalar and pseudo-scalar Higgs bosons to next-to-next-to-leading order at hadron colliders. JHEP.

[39] Catani S, Trentadue L (1989). Resummation of the QCD perturbative series for hard processes. Nucl. Phys. B.

[40] Sterman GF (1987). Summation of large corrections to short distance hadronic cross-sections. Nucl. Phys. B.

[41] H. Contopanagos, E. Laenen, G.F. Sterman, Sudakov factorization and resummation. Nucl. Phys. B **484**, 303–330 (1997). arXiv:hep-ph/9604313

[42] S. Forte, G. Ridolfi, Renormalization group approach to soft gluon resummation. Nucl. Phys. B **650**, 229–270 (2003). arXiv:hep-ph/0209154

[43] S. Catani, M.L. Mangano, P. Nason, L. Trentadue, The Resummation of soft gluons in hadronic collisions. Nucl. Phys. B **478**, 273–310 (1996). arXiv:hep-ph/9604351

[44] Ahrens V, Becher T, Neubert M, Yang LL (2009). Renormalization-group improved prediction for Higgs production at Hadron colliders. Eur. Phys. J. C.

[45] T. Becher, M. Neubert, B.D. Pecjak, Factorization and momentum-space resummation in deep-inelastic scattering. JHEP **0701**, 076 (2007). arXiv:hep-ph/0607228

[46] Becher T, Neubert M, Xu G (2008). Dynamical threshold enhancement and resummation in Drell–Yan production. JHEP.

[47] T. Becher, M. Neubert, Threshold resummation in momentum space from effective field theory. Phys. Rev. Lett. **97**, 082001 (2006). arXiv:hep-ph/0605050 10.1103/PhysRevLett.97.082001 17026292

[48] M. Bonvini, S. Forte, G. Ridolfi, L. Rottoli, Resummation prescriptions and ambiguities in SCET vs. direct QCD: Higgs production as a case study. *JHEP* **1501**, 046 (2015). arXiv:1409.0864

[49] Sterman G, Zeng M (2014). Quantifying comparisons of threshold resummations. JHEP.

[50] Bonvini M, Forte S, Ghezzi M, Ridolfi G (2012). Threshold resummation in SCET vs. perturbative QCD: an analytic comparison. Nucl. Phys..

[51] Bonvini M, Forte S, Ghezzi M, Ridolfi G (2013). The scale of soft resummation in SCET vs perturbative QCD. Nucl. Phys. Proc. Suppl..

[52] S. Moch, J. Vermaseren, A. Vogt, Higher-order corrections in threshold resummation. Nucl. Phys. B **726**, 317–335 (2005). arXiv:hep-ph/0506288

[53] Li Y, von Manteuffel A, Schabinger RM, Zhu HX (2015). Soft-virtual corrections to Higgs production at N$$^3$$ LO. Phys. Rev. D.

[54] Ahmed T, Gehrmann T, Mathews P, Rana N, Ravindran V (2015). Pseudo-scalar form factors at three loops in QCD. JHEP.

[55] Ahrens V, Becher T, Neubert M, Yang LL (2009). Origin of the large perturbative corrections to Higgs production at Hadron colliders. Phys. Rev. D.

[56] Parisi G (1980). Summing large perturbative corrections in QCD. Phys. Lett. B.

[57] Magnea L, Sterman GF (1990). Analytic continuation of the Sudakov form-factor in QCD. Phys. Rev. D.

[58] A.P. Bakulev, A.V. Radyushkin, N.G. Stefanis, Form-factors and QCD in space - like and time-like region. Phys. Rev. D **62**, 113001 (2000). arXiv:hep-ph/0005085

[59] T.O. Eynck, E. Laenen, L. Magnea, Exponentiation of the Drell–Yan cross-section near partonic threshold in the DIS and MS-bar schemes. JHEP **06**, 057 (2003). arXiv:hep-ph/0305179

[60] I.W. Stewart, F.J. Tackmann, J.R. Walsh, S. Zuberi, Jet $$p_T$$ resummation in Higgs production at $$NNLL^{\prime }+NNLO$$. Phys. Rev. D **89**(5), 054001 (2014). arXiv:1307.1808

[61] Ball RD, Bonvini M, Forte S, Marzani S, Ridolfi G (2013). Higgs production in gluon fusion beyond NNLO. Nucl. Phys. B.

[62] de Florian D, Mazzitelli J, Moch S, Vogt A (2014). Approximate N$$^{3}$$ LO Higgs-boson production cross section using physical-kernel constraints. JHEP.

[63] Anastasiou C, Duhr C, Dulat F, Furlan E, Gehrmann T, Herzog F, Mistlberger B (2015). Higgs boson gluon-fusion production beyond threshold in N$$^{3}$$ LO QCD. JHEP.

[64] Anastasiou C, Duhr C, Dulat F, Furlan E, Gehrmann T, Herzog F, Mistlberger B (2014). Higgs boson gluon-fusion production at threshold in N$$^3$$ LO QCD. Phys. Lett. B.

[65] Catani S, Cieri L, de Florian D, Ferrera G, Grazzini M (2014). Threshold resummation at N$$^3$$ LL accuracy and soft-virtual cross sections at N$$^3$$ LO. Nucl. Phys. B.

[66] Laenen E, Magnea L, Stavenga G (2008). On next-to-eikonal corrections to threshold resummation for the Drell–Yan and DIS cross sections. Phys. Lett. B.

[67] Laenen E, Stavenga G, White CD (2009). Path integral approach to eikonal and next-to-eikonal exponentiation. JHEP.

[68] Laenen E, Magnea L, Stavenga G, White CD (2011). Next-to-eikonal corrections to soft gluon radiation: a diagrammatic approach. JHEP.

[69] Grunberg G, Ravindran V (2009). On threshold resummation beyond leading 1-x order. JHEP.

[70] Bonocore D, Laenen E, Magnea L, Vernazza L, White CD (2015). The method of regions and next-to-soft corrections in Drell–Yan production. Phys. Lett. B.

[71] Bonocore D, Laenen E, Magnea L, Melville S, Vernazza L, White CD (2015). A factorization approach to next-to-leading-power threshold logarithms. JHEP.

[72] Caola F, Marzani S (2011). Finite fermion mass effects in pseudoscalar Higgs production via gluon–gluon fusion. Phys. Lett. B.

[73] F. Herzog, B. Mistlberger, The Soft-Virtual Higgs Cross-section at N3LO and the Convergence of the Threshold Expansion. In *Proceedings, 49th Rencontres de Moriond on QCD and High Energy Interactions: La Thuile, Italy, March 22–29, 2014*, pp. 57–60 (2014). arXiv:1405.5685

[74] NNPDF Collaboration, R.D. Ball et al., Parton distributions for the LHC Run II. JHEP **04**, 040 (2015). arXiv:1410.8849

[75] Buckley A, Ferrando J, Lloyd S, Nordström K, Page B, Rüfenacht M, Schönherr M, Watt G (2015). LHAPDF6: parton density access in the LHC precision era. Eur. Phys. J. C.

[76] Bonvini M, Ball RD, Forte S, Marzani S, Ridolfi G (2014). Updated Higgs cross section at approximate N$$^3$$ LO. J. Phys..

[77] http://www.ge.infn.it/~bonvini/higgs/

[78] http://www.ge.infn.it/~bonvini/troll/

[79] ATLAS Collaboration, M. Aaboud et al., Search for minimal supersymmetric standard model Higgs bosons $$H/A$$ and for a $$Z^{\prime }$$ boson in the $$\tau \tau $$ final state produced in $$pp$$ collisions at $$\sqrt{s}=13$$ TeV with the ATLAS detector. Eur. Phys. J. C **76**(11), 585 (2016). arXiv:1608.00890 10.1140/epjc/s10052-016-4400-6 PMC5335571 28316491

[80] ATLAS Collaboration, T.A. collaboration, Search for minimal supersymmetric standard model Higgs bosons $$H/A$$ in the $$\tau \tau $$ final state in up to 13.3 fb$$^{-1}$$ of pp collisions at $$\sqrt{s}=13$$ TeV with the ATLAS detector

[81] Djouadi A, Maiani L, Moreau G, Polosa A, Quevillon J, Riquer V (2013). The post-Higgs MSSM scenario: Habemus MSSM?. Eur. Phys. J. C.

[82] ATLAS Collaboration, Search for resonances in diphoton events with the ATLAS detector at $$\sqrt{s}$$ = 13 TeV, ATLAS-CONF-2016-018

[83] CMS Collaboration, Search for new physics in high mass diphoton events in $$3.3~{\rm fb}^{-1}$$ of proton-proton collisions at $$\sqrt{s}=13~{\rm TeV}$$ and combined interpretation of searches at $$8~{\rm TeV}$$ and $$13~{\rm TeV}$$, CMS-PAS-EXO-16-018

[84] Bonciani R, Degrassi G, Vicini A (2007). Scalar particle contribution to Higgs production via gluon fusion at NLO. JHEP.

[85] C. Anastasiou, C. Duhr, F. Dulat, E. Furlan, T. Gehrmann, F. Herzog, A. Lazopoulos, B. Mistlberger, CP-even scalar boson production via gluon fusion at the LHC. arXiv:1605.05761

[86] R.V. Harlander, S. Liebler, H. Mantler, SusHi: a program for the calculation of Higgs production in gluon fusion and bottom-quark annihilation in the Standard Model and the MSSM. Comput. Phys. Commun. **184**, 1605–1617 (2013). arXiv:1212.3249

[87] R.V. Harlander, S. Liebler, H. Mantler, SusHi Bento: beyond NNLO and the heavy-top limit. arXiv:1605.03190

[88] Harlander RV, Mantler H, Marzani S, Ozeren KJ (2010). Higgs production in gluon fusion at next-to-next-to-leading order QCD for finite top mass. Eur. Phys. J. C.

[89] Pak A, Rogal M, Steinhauser M (2010). Finite top quark mass effects in NNLO Higgs boson production at LHC. JHEP.

